# Availability of primary care physicians and hepatocellular carcinoma‐related mortality in the United States

**DOI:** 10.1002/jgf2.679

**Published:** 2024-02-21

**Authors:** Daniyal Raza, Udhayvir Singh Grewal

**Affiliations:** ^1^ Department of Medicine Louisiana State University Health Sciences Center Shreveport Louisiana USA; ^2^ Division of Hematology, Oncology, Blood and Marrow Transplantation University of Iowa Hospitals and Clinics Iowa City Iowa USA

## Abstract

Hepatocellular carcinoma (HCC) is the fifth leading cause of cancer worldwide and majority cases are diagnosed at an intermediate or advanced stage. Per our analysis, greater availability of primary care physicians correlates with lower HCC‐related mortality. Our results underscore the need for efforts to expand access to primary care among all populations, especially African Americans, to improve overall HCC‐related outcomes.

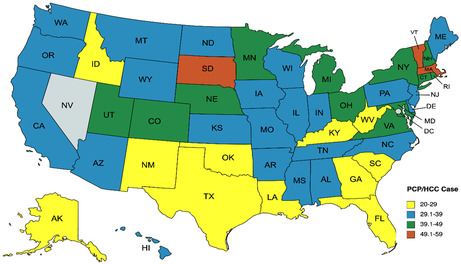

To the Editor,

Hepatocellular carcinoma (HCC) ranks as the fifth leading cause of cancer worldwide and majority cases are diagnosed at an intermediate or advanced stage. Additionally, fewer than 20% of patients with cirrhosis undergo consistent surveillance for HCC.^1^ We sought to study the impact of presence of primary care physicians (PCPs) on the overall and race‐specific mortality for HCC in the US.

Data regarding the crude incidence of HCC across all 50 states and the District of Columbia (D.C.) for the year 2019 were gathered from the Center for Disease Control (CDC) WONDER database. Information on the number of actively practicing PCPs in these areas was acquired from the Association of American Medical Colleges (AAMC) 2021 State Physician Workforce Data Report (2020). Our analysis focused on examining the correlation between the ratio of PCPs to HCC cases (referred to as PCP‐per‐case ratio) and the age‐adjusted mortality rate (AAMR) of HCC across 51 states, excluding Nevada due to data unavailability, for the year 2019. Additionally, a comparative analysis of mortality rates between Caucasian and African American (A.A.) populations in 2019 was conducted, with data available from only 31 states.

The median AAMR for HCC was 4.35 (IQR 3.7, 5.2) per 100,000 population. AAMR was higher among A.A. than Caucasian populations (*t* = 6.44, *p* < 0.001). PCP‐per‐case ratio was significantly higher among A.A. compared to Caucasian populations (*t* = +6.32, *p* < 0.0001). We found that the higher state‐wide PCP‐per‐case ratio correlated with lower HCC‐related mortality (*r* = −0.375, *p* = 0.007). A higher PCP‐per‐case ratio correlated with lower AAMR among Caucasians (*r* = −0.6973, *p* < 0.001). However, the PCP‐per‐case ratio did not correlate with AAMR among A.A. (*r* = −0.027, *p* = 0.88). Figure 1 shows the geographical distribution of PCP‐per‐HCC cases for all 50 U.S. states except Nevada.

**FIGURE 1:**
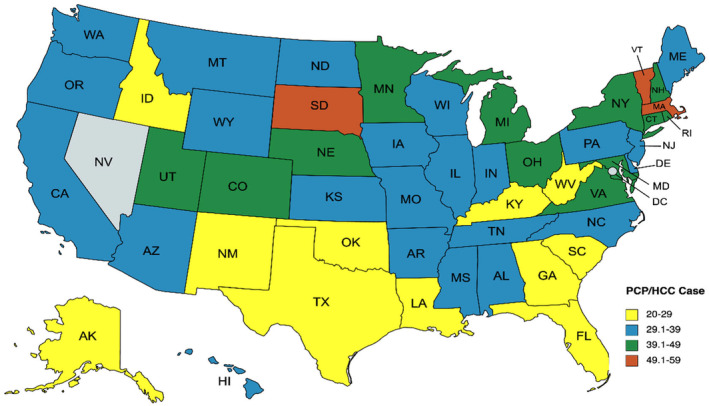
Geographical distribution of primary care physician (PCP) per hepatocellular carcinoma (HCC) case ratios for all 50 U.S. states (data for Nevada were missing).

Per our analysis, there is a correlation suggesting that higher availability of PCPs correlates with a lower mortality related to HCC. Despite greater PCP availability among A.A. populations (higher PCP‐per‐case ratio), AAMR was higher than Caucasians. These findings underpin a potential lack of access to primary care despite adequate availability. A.A. with HCC have been historically known to present with advanced disease and have poorer survival.^2^ Based on the current analysis, it is possible that poorer access to PCPs may be contributing to lower rates of screening and surveillance among A.A. These results are consistent with prior studies indicating the relative underutilization of imaging‐based HCC surveillance among AA populations.^3^ Various interventions could potentially enhance the rates of HCC surveillance at the primary care level such as automatic reminders to PCPs or nurse or pharmacist‐based protocols to allow expansion of ancillary support to PCPs.^4^ Additionally, improving access to subspecialist‐based HCC surveillance may also lead to an overall improvement in adherence to HCC surveillance recommendations.^5^

In conclusion, improving access to primary care physicians may contribute to improvement in overall outcomes related to HCC. It is also possible that poorer access to PCPs may be contributing to lower rates of screening and surveillance among A.A. Our results highlight the importance of efforts geared toward the expansion of access to primary care among all populations, especially A.A., to improve overall outcomes related to HCC.

## CONFLICT OF INTEREST STATEMENT

The authors have stated explicitly that there are no conflicts of interest in connection with this article.
